# Intermittent Energy Restriction for Adolescents With Obesity: The Fast Track to Health Randomized Clinical Trial

**DOI:** 10.1001/jamapediatrics.2024.2869

**Published:** 2024-08-26

**Authors:** Natalie B. Lister, Louise A. Baur, Eve T. House, Shirley Alexander, Justin Brown, Clare E. Collins, Christopher T. Cowell, Kaitlin Day, Sarah P. Garnett, Megan L. Gow, Alicia M. Grunseit, Maddison Henderson, Mary-Kate Inkster, Cathy Kwok, Sarah Lang, Susan J. Paxton, Helen Truby, Krista A. Varady, Hiba Jebeile

**Affiliations:** 1 Faculty of Medicine and Health, Sydney Medical School, The University of Sydney, Westmead, New South Wales, Australia; 2 Institute of Endocrinology and Diabetes, The Children’s Hospital at Westmead, Westmead, New South Wales, Australia; 3 Weight Management Services, The Children’s Hospital at Westmead, Westmead, New South Wales, Australia; 4 Department of Paediatric Endocrinology and Diabetes, Monash Children’s Hospital, Clayton, Victoria, Australia; 5 Department of Paediatrics, Monash University, Clayton, Victoria, Australia; 6 School of Health Sciences, College of Health, Medicine and Wellbeing, The University of Newcastle, Callaghan, New South Wales, Australia; 7 Food and Nutrition Research Program, Hunter Medical Research Institute, New Lambton Heights, New South Wales, Australia; 8 Kids Research, The Children’s Hospital at Westmead, Westmead, New South Wales, Australia; 9 School of Agriculture, Food and Ecosystem Sciences, The University of Melbourne, Melbourne, Victoria, Australia; 10 Department of Nutrition, Dietetics & Food, Monash University, Melbourne, Victoria, Australia; 11 Department of Nutrition and Dietetics, The Children’s Hospital at Westmead, Westmead, New South Wales, Australia; 12 School of Psychology and Public Health, La Trobe University, Melbourne, Victoria, Australia; 13 School of Primary and Allied Health Care, Monash University, Victoria, Australia; 14 School of Human Movement and Nutrition Sciences, University of Queensland, Queensland, Australia; 15 Department of Kinesiology and Nutrition, University of Illinois, Chicago

## Abstract

**Importance:**

Adolescent obesity requires effective and accessible treatment. Intensive dietary interventions have the potential to be used as adjunctive therapy for behavioral weight management.

**Objective:**

To examine the effectiveness of 2 diet therapies, delivered as part of an intensive behavioral weight management intervention, in adolescents with metabolic complications associated with obesity.

**Design, Setting, and Participants:**

This multisite, 52-week randomized clinical trial was conducted from January 31, 2018, to March 31, 2023, at 2 tertiary pediatric centers in Australia. Adolescents (aged 13-17 years) with obesity and 1 or more associated complications were included.

**Interventions:**

Intensive behavioral interventions, delivered by a multidisciplinary team, comparing intermittent energy restriction (IER) or continuous energy restriction (CER), with 3 phases: very low-energy diet (weeks 0-4), intensive intervention (weeks 5-16), and continued intervention and/or maintenance (weeks 17-52).

**Main Outcomes and Measures:**

The primary outcome was body mass index (BMI) *z* score at 52 weeks in the IER vs CER group. Anthropometry, body composition, and cardiometabolic health were assessed at baseline and 52 weeks. The BMI *z* score and percentiles were determined using Centers for Disease Control and Prevention growth charts. Insulin resistance, dyslipidemia, and elevated hepatic function were assessed.

**Results:**

A total of 141 adolescents (median [IQR] age, 14.8 [12.9-17.9] years; 71 male [50.4%]) were enrolled, 71 in the IER group and 70 in the CER group, and 97 (68.8%) completed the intervention, 43 in the IER group and 54 in the CER group. At week 52, both groups had reduced BMI *z* scores (estimated marginal mean change, −0.28 [95% CI, −0.37 to −0.20] for IER and −0.28 [95% CI, −0.36 to −0.20] for CER) and reduced BMI expressed as a percentage of the 95th percentile (estimated marginal mean change, −9.56 [95% CI, −12.36 to −6.83] for IER and −9.23 [95% CI, −11.82 to −6.64] for CER). No differences were found in body composition or cardiometabolic outcomes between the groups. Both groups had a reduction in the occurrence of insulin resistance (from 52 of 68 [76.5%] to 32 of 56 [57.1%] in the IER group and from 59 of 68 [86.8%] to 31 of 60 [57.1%] in the CER group) at week 16; however, at week 52, this effect was observed in the CER group only (from 59 of 68 [86.7%] to 30 of 49 [61.2%]). The occurrence of dyslipidemia was unchanged between baseline and week 52 (60 of 137 [42.6%] and 37 of 87 [42.5%], respectively), with a small improvement in occurrence of impaired hepatic function tests (37 of 139 [27.0%] and 15 of 87 [17.2%], respectively). No differences were found in dyslipidemia or hepatic function between groups.

**Conclusions and Relevance:**

These findings suggest that for adolescents with obesity-associated complications, IER can be incorporated into a behavioral weight management program, providing an option in addition to CER and offering participants more choice.

**Trial Registration:**

http://anzctr.org.au Identifier: ACTRN12617001630303

## Key Points

**Question:**

In adolescents with obesity, does intermittent energy restriction lead to lower body mass index *z* score and cardiometabolic risk after 52 weeks compared with continuous energy restriction?

**Findings:**

In this randomized clinical trial of 141 adolescents with obesity, no differences were found in improvements in body composition or cardiometabolic health for those receiving intermittent vs continuous energy restriction as part of an intensive behavioral weight management program after 52 weeks.

**Meaning:**

These findings suggest that intermittent and continuous energy restriction delivered as part of an intensive behavioral weight management program may both be beneficial options to improve weight and cardiometabolic outcomes for adolescents.

## Teaser

This randomized clinical trial of adolescents with obesity assesses whether intermittent energy restriction compared with continuous energy restriction leads to a lower body mass index *z* score and improved body composition and cardiometabolic profile.

## Introduction

Obesity is predicted to affect more than 254 million children and adolescents (aged 5-19 years) worldwide by 2030.^1^ Complications from adolescent obesity can occur in the immediate or long term and may include metabolic, cardiovascular, pulmonary, musculoskeletal, or psychosocial conditions.^2^ Behavioral weight management is foundational for treatment of adolescent obesity^4^ and is recommended in clinical practice guidelines.^5^ However, the efficacy of behavioral interventions is limited compared with pharmacotherapy^7^ or bariatric surgery,^8^ which are recommended as adjunctive therapies for adolescents with severe obesity.^5^ Intervention effectiveness is important for long-term outcomes, with evidence suggesting early weight loss is associated with better longer-term outcomes.^9^ Hence, effective and accessible intervention options are needed. Given that pharmacologic and surgical interventions are subject to cost and accessibility issues, there is a potential for intensive dietary interventions as adjunctive therapeutic alternatives for some individuals with comorbidities.^4^

Intensive dietary interventions differ from conventional dietary advice included within behavioral treatments and aim to markedly reduce total energy intake through use of very low-energy diets (VLEDs)^10^ or intermittent energy restriction (IER).^11^ These interventions may be used when conventional treatment is unsuccessful, when rapid and substantial weight loss is required due to comorbidities,^13^ or when pharmacotherapy or surgery is unavailable. A 2019 systematic review of VLEDs found a mean weight reduction of 5.3 kg at latest follow-up (5.0-14.5 months) in children and adolescents with obesity.^14^ In adults, IER was equally effective as continuous energy restriction (CER) in the medium to longer term (6-12 months).^15^ In a feasibility study of adolescents, IER was considered an acceptable intervention^16^; however, effectiveness compared with CER has not been tested.

This study aimed to compare effectiveness of 2 diet therapies, delivered as part of an intensive behavioral weight management intervention by a multidisciplinary team, in adolescents with metabolic complications associated with obesity. The diet therapies were a VLED followed by IER or CER. We hypothesized that, compared with CER, IER would be acceptable to adolescents and their families, leading to a lower body mass index (BMI) *z* score and improved body composition and cardiometabolic profile after 52 weeks of intervention.

## Methods

This multisite, parallel, controlled randomized clinical trial was conducted in Australia at The Children’s Hospital at Westmead (site 1) and Monash Children’s Hospital (site 2) between January 31, 2018, and March 31, 2023. The study was approved by the Sydney Children’s Hospitals Network Human Research Ethics Committee and reported according to the Consolidated Standards of Reporting Trials (CONSORT) reporting guideline for randomized clinical trials.^17^ The protocol has been previously published^18^ and is described briefly here. The trial protocol is given in Supplement 1. Major protocol changes are listed in eTable 1 in Supplement 2. An independently chaired data safety monitoring committee oversaw study safety.^18^

### Participants and Recruitment

We sought to recruit 186 adolescents (aged 13-17 years) with obesity (defined as adult BMI greater than or equal to 30 [calculated as weight in kilograms divided by height in meters squared][Bibr poi240051r19]) and at least 1 cardiometabolic complication, including prediabetes, insulin resistance, acanthosis nigricans, hypertension, low high-density lipoprotein cholesterol (HDL-C) level, high level of triglycerides, elevated alanine transaminase or γ-glutamyltransferase level, or a diagnosis of polycystic ovary syndrome.^18^ Exclusion criteria were secondary obesity, significant intellectual disability, medical or psychiatric illness, type 2 diabetes, currently undergoing treatment for a clinical eating disorder, pregnancy or planning pregnancy, taking medications that affect weight in the short term (excluding metformin), adolescent or parent unable to communicate in English, current enrollment in a weight management program, or having a BMI greater than 45.

Participants were recruited via health professionals or self-referral. After initial telephone screening, participants underwent an in-person eligibility screening, including a medical assessment and screening for clinical depression (using the Center for Epidemiologic Studies Depression Scale–Revised 10-item version for adolescents [CESD-R-10][Bibr poi240051r20]) and eating disorders (using the Eating Disorder Examination Questionnaire [EDE-Q][Bibr poi240051r21]). Adolescents meeting prespecified cut points on the CESD-R-10 and/or EDE-Q were reviewed by a clinical psychologist or pediatrician. Further information describing eating disorder and depression screening and monitoring are published.^18^

### Randomization and Masking

After parents provided informed written consent and adolescents agreed to participate, a computer randomization schedule from The University of Sydney Clinical Trials Centre allocated participants to 1 of 2 treatment arms. Randomization occurred via minimization and was stratified by age (13-14 years or 15-17 years), sex, BMI (≥30 to <35 or 35 to 45), and study site. Information on ethnicity was collected as per standard practice for clinical trials. Siblings were randomized as dyads using forced allocation. Participants were informed of their allocated dietary intervention at the commencement of the intervention.

### Intervention

Detailed meal plans tailored to meet dietary needs, food preferences, and lifestyle were provided. Participants were not blinded to the intervention. Interventions were delivered by trained study dietitians. Participants were reviewed by a study pediatrician at week 16, and additional reviews by the study pediatrician, dietitian, or clinical psychologist were conducted if required.

#### Phase 1

For the first 4 weeks all participants were prescribed a VLED of 3350 kJ/d (800 kcal/d), using micronutrient complete meal replacement products (Optifast, Nestlé) for 3 to 4 meals per day and low-energy food. Meal replacement products were provided at no cost. Participants met with the dietitian weekly during phase 1, with telephone support during the first week.

#### Phase 2 

At week 5, participants transitioned to either the IER or CER intervention. The IER intervention prescribed 2500 to 2950 kJ (approximately 600-700 kcal) 3 days per week; low-energy days were alternated with 4 days per week of healthy eating based on the Australian Dietary Guidelines with no energy prescription. The CER intervention was a daily energy prescription based on age: 6000 to 7000 kJ/d (1430-1670 kcal/d) for those aged 13 to 14 years and 7000 to 8000 kJ/d (1670-1900 kcal/d) for those aged 15 to 17 years. All participants were provided with a multivitamin containing fish oil and instructed to take 1 multivitamin daily. Participants met with the dietitian at weeks 6, 9, 12, and 16 with support provided via telephone, text message, or email at weeks 8, 11, and 14.

#### Phase 3 

Phase 3 commenced at week 17. There was no change to the dietary intervention unless participants reached their personal goal weight or calculated healthy body weight (ie, adult BMI of 25 using International Obesity Task Force age- and sex-adjusted BMI cutoffs; personal goal weights could not be lower than healthy body weight). Participants who reached their goal weight were transitioned to a weight maintenance dietary plan. Participants met with the dietitian less frequently during phase 3, at weeks 20, 26, 36, and 52, with support provided via telephone, text message, or email at weeks 18, 24, 28, 42, and 48. On completion, participants were provided with referral avenues for ongoing support, and strategies for weight maintenance were discussed.

#### Effects of COVID-19

During the COVID-19 pandemic, recruitment was halted at site 1 for 4 months and site 2 for 7 months due to local government health directives. During lockdowns, intervention delivery continued face to face for visits at baseline and weeks 4, 16, and 52, with most dietetic reviews occurring via telehealth unless face-to-face reviews were clinically indicated. Additional visits were offered by telehealth or face-to-face appointments.

### Outcome Assessments

Data on demographics and medical history were collected at baseline during clinical review. Outcomes were measured blinded by trained assessors using standard procedures^18^ at baseline and weeks 4, 16, and 52. The primary outcome was BMI *z* score (BMI*z*) at 52 weeks in the IER vs CER group. Weight, height, and waist circumference were measured to calculate BMI, BMI*z*,^23^ BMI expressed as a percentage of the 95th percentile (BMI95),^24^ and waist to height ratio. Elevated blood pressure was defined as systolic blood pressure of 120 mm Hg or higher or a diastolic blood pressure of 80 mm Hg or higher.^25^ Blood drawn at baseline, week 16, and week 52 was analyzed in accredited pathology laboratories for triglycerides, HDL-C, glucose, insulin, alanine aminotransferase, γ-glutamyl transferase, glucose, and insulin levels. Dyslipidemia was defined as levels of triglycerides greater than 150 mg/dL (to convert to millimoles per liter, multiply by 0.0113) and HDL-C levels less than 40 mg/dL (to convert to millimoles per liter, multiply by 0.0259). An elevated alanine aminotransferase or γ-glutamyl transferase level was defined as 1.5 times or more than the upper limit of 30 U/L (to convert to microkatals per liter, multiply by 0.0167).^26^ Insulin resistance was defined as a fasting insulin-glucose ratio greater than 20.^27^ Elevated fasting glucose was defined as 100 to 124 mg/dL (to convert to millimoles per liter, multiply by 0.0555).^29^ Body composition (fat mass and fat-free mass) was measured using dual-energy x-ray absorptiometry (DXA; ProdigyLunar-GE DXA at site 1 and Lunar iDXA [GE HealthCare] at site 2). Fat mass index (total fat mass divided by height squared) and fat-free mass index (fat-free mass divided by height squared) were calculated.^30^ Adverse events, defined as any untoward medical occurrences that did not necessarily have a causal relationship with the intervention,^31^ were recorded at each visit.

### Statistical Analysis

Analysis was conducted masked to treatment allocation, according to a prespecified plan. Sample size was based on change in BMI*z* between intervention groups at week 52.^18^ The mean (SD) difference between groups was estimated to be 0.12 (0.24). Assuming 80% power and a 2-sided significance level of *P* < .05, a sample size of 65 adolescents per group (total of 130) was required. Allowing for a 30% attrition rate, we aimed to recruit 186 adolescents. Statistical analysis was performed using SPSS Statistics, version 28.0 (IBM Inc). All participant data were retained. Linear mixed models were used to estimate the change in outcomes at baseline, week 4, week 16, and week 52 consistent with intention to treat for longitudinal analysis (eMethods in Supplement 2). Nine sibling pairs were recruited. Analyses were repeated removing 1 participant from each sibling pair with no differences in results, so all data were retained in analyses. No adjustments were made for multiple comparisons.

## Results

**Figure 1.**
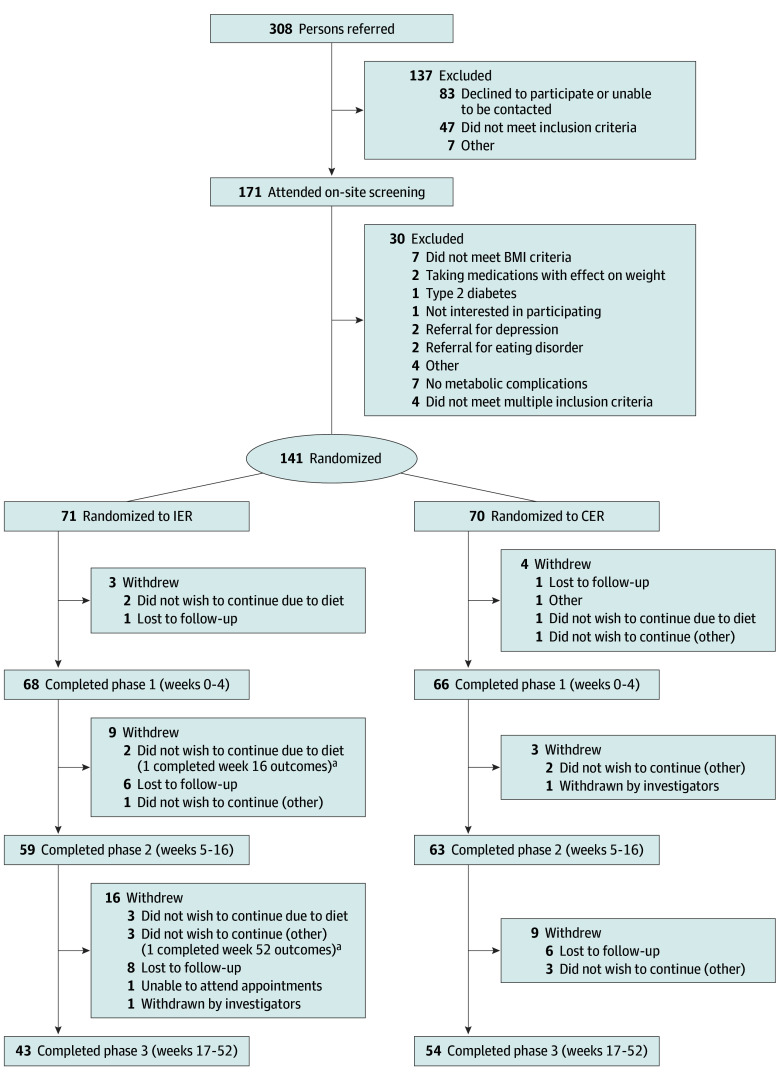
CONSORT Diagram Showing Participant Flow in the Study BMI indicates body mass index (calculated as weight in kilograms divided by height in meters squared); CER, continuous energy restriction; IER, intermittent energy restriction. ^a^Two participants withdrew from IER treatment but returned for the next measurement visit (1 at 16 weeks and 1 at 52 weeks).

Of the 208 adolescents screened, 141 (median [IQR] age, 14.8 [12.9-17.9] years; 70 female [49.6%] and 71 male [50.4%]) were enrolled, 71 in the IER group and 70 in the CER group, and 97 (68.8%) completed the intervention, 43 in the IER group and 54 in the CER group (Figure 1 and Table 1). Fifty participants (35.5%) were enrolled before 2019 and completed the intervention before the COVID-19 pandemic, whereas 70 participants (49.6%) were recruited after 2020. Delays due to COVID-19 resulted in an extension of recruitment by 2 years (originally planned to finish November 2020), and the trial closed to recruitment due to time and funding constraints. A total of 117 of the 141 participants (82.9%) were born in Australia, 6 (4.3%) in India, 2 (1.4%) in Vietnam, and 9 (6.4%) in other countries (not further specified), with 2 (1.4%) identifying as Aboriginal or Torres Strait Islander (other ethnicities were not collected). Seven participants declined to respond. However, of the 141 patients, 67 (47.5%) reported 1 or both parents were not born in Australia (eTable 2 in Supplement 2), and 57 (40.4%) reported speaking a language other than English at home. Most participants reported a family history of obesity (97 [68.8%]) or diabetes (117 [83.0%]). Medications taken included metformin (20 [14.2%]) and asthma-relieving medications (14 [9.9%]). There were no differences in demographics or baseline characteristics between participants who withdrew and those who completed the intervention. More withdrew from the IER group (28 of 71 [39.4%]) compared with the CER group (16 of 70 [22.9%]). Completers had greater weight loss at week 4 (mean [SD] BMI*z* change, −0.22 [0.12] compared with −0.17 [0.09] for participants who withdrew). There were no differences in sex between withdrawals and completers in either intervention group. During phase 1, 3 participants withdrew because they did not wish to continue with the VLED (Figure 1). The most common reasons for nonparticipation during phases 2 and 3 were loss to follow-up (14 in the IER group and 6 in the CER group) and not wishing to continue the study diet (5 in the IER group). There were no differences between dietitian attendance between groups, and 106 participants (75.2%) attended more than 9 scheduled visits.

**Table 1.** Baseline Characteristics of the Participants

Characteristic	All participants (N = 141)	IER group (n = 71)	CER group (n = 70)
Age, median (range), y	14.8 (12.9-17.9)	14.8 (12.9-17.9)	14.8 (12.9-17.8)
Sex, No. (%)			
Male	71 (50.4)	35 (49.3)	36 (51.4)
Female	70 (49.6)	36 (50.7)	34 (48.6)
Aboriginal or Torres Strait Islander	2 (1.4)	1 (1.4)	1 (1.4)
Anthropometry, mean (SD)			
Weight, kg	100.42 (16.50)	97.71 (15.09)	103.09 (17.61)
Weight *z* score	2.55 (0.52)	2.47 (0.51)	2.61 (0.53)
Height, cm	168.16 (9.25)	167.31 (9.90)	168.98 (8.59)
Height *z* score	0.51 (1.06)	0.44 (1.18)	0.58 (0.93)
Waist circumference, cm	107.45 (11.61)	105.61(11.00)	109.44 (12.01)
Waist to height ratio	0.64 (0.06)	0.63 (0.06)	0.65 (0.06)
BMI	35.39 (4.17)	34.83 (3.91)	35.95 (4.40)
BMI *z* score	2.40 (0.46)	2.34 (0.43)	2.45 (0.49)
BMI95	130 (15)	128 (14)	132 (16)
Waist to height ratio ≥0.5, No. (%)	139 (98.6)	70 (98.6)	69 (98.6)
Body composition, mean (SD) (N = 140; IER, n = 71; CER, n = 69)			
FMI	16.18 (3.25)	15.95 (3.06)	16.41 (3.45)
FFMI	18.67 (1.85)	18.41 (1.80)	18.94 (1.86)
Body fat percentage	46.09 (5.23)	46.12 (5.05)	46.06 (5.44)
Cardiometabolic profile			
Systolic blood pressure, mean (SD), mm Hg	119.5 (10.5)	118.8 (10.5)	120.3 (10.5)
Diastolic blood pressure, mean (SD), mm Hg	68.1 (9.3)	67.2 (9.0)	69.0 (9.5)
Systolic blood pressure >95th percentile, No. (%)	17 (12.1)	10 (14.1)	7 (10)
Diastolic blood pressure >95th percentile, No. (%)	17 (12.1)	10 (14.1)	7 (10)
Total cholesterol, median (95% CI), mg/dL (N = 128; IER, n = 66; CER, n = 62)	166 (166-178)	170 (162-181)	166 (162-181)
HDL-C, median (95% CI), mg/dL (N = 128; IER, n = 66; CER, n = 62)	42 (42-46)	42 (42-46)	42 (42-46)
LDL-C, median (95% CI), mg/dL (N = 128; IER, n = 66; CER, n = 62)	112 (112-124)	116 (108-127)	108 (108-143)
LDL-C:HDL-C ratio, median (95% CI) (N = 128; IER, n = 66; CER, n = 62)	2.7 (2.6-2.9)	2.7 (2.6-2.8)	2.6 (2.5-3.0)
Triglycerides, median (95% CI), mg/dL (N = 128; IER, n = 66; CER, n = 62)	115 (115-133)	115 (106-133)	115 (115-150)
Dyslipidemia, No. (%) (N = 137; IER, n = 70; CER, n = 67)	60 (42.6)	31 (43.7)	29 (41.4)
Alanine transaminase, median (95% CI), U/L (N = 135; IER, n = 69; CER, n = 66)	29.0 (34.2-36.4)	31.0 (34.5-48.8)	27.5 (27.6-63.3)
γ-Glutamyl transpeptidase, median (95% CI), U/L (N = 135; IER, n = 69; CER, n = 66)	22.0 (24.1-29.8)	22.0 (23.3-31.2)	23.0 (22.4-30.8)
Elevated liver enzymes, No. (%) (N = 139; IER, n = 70; CER, n = 69)	37 (27.0)	24 (34.3)	13 (18.8)
Insulin, median (95% CI), μIU/mL (N = 137; IER, n = 68; CER, n = 69)	22 (23-29)	24 (23-32)	20 (22-28)
Insulin resistance, No. (%) (N = 136; IER, n = 68; CER, n = 68)	111 (81.6)	52 (76.5)	59 (86.8)
Fasting glucose, median (95% CI), mg/dL (N = 138, IER, n = 70; CER, n = 68)	88 (88-90)	86 (86-90)	90 (88-92)
Elevated fasting glucose of 100.90-124.32 mg/dL, No. (%) (N = 138; IER, n = 70; CER, n = 68)	11 (8.0)	5 (7.0)	6 (8.6)

Abbreviations: BMI, body mass index (calculated as weight in kilograms divided by height in meters squared); BMI95, BMI expressed as a percentage of the 95th percentile; CER, continuous energy restriction; FMI, fat mass index; FFMI, fat-free mass index; HDL-C, high-density lipoprotein cholesterol; IER, intermittent energy restriction; LDL-C, low-density lipoprotein cholesterol.

SI conversion factors: To convert cholesterol to millimoles per liter, multiply by 0.0259; triglycerides to millimoles per liter, multiply by 0.0113; alanine aminotransferase and γ-glutamyl transpeptidase to microkatals per liter, multiply by 0.0167; insulin to picomoles per liter, multiply by 6.945; and glucose to millimoles per liter, multiply by 0.0555.

### Weight and Body Composition Outcomes

**Figure 2.**
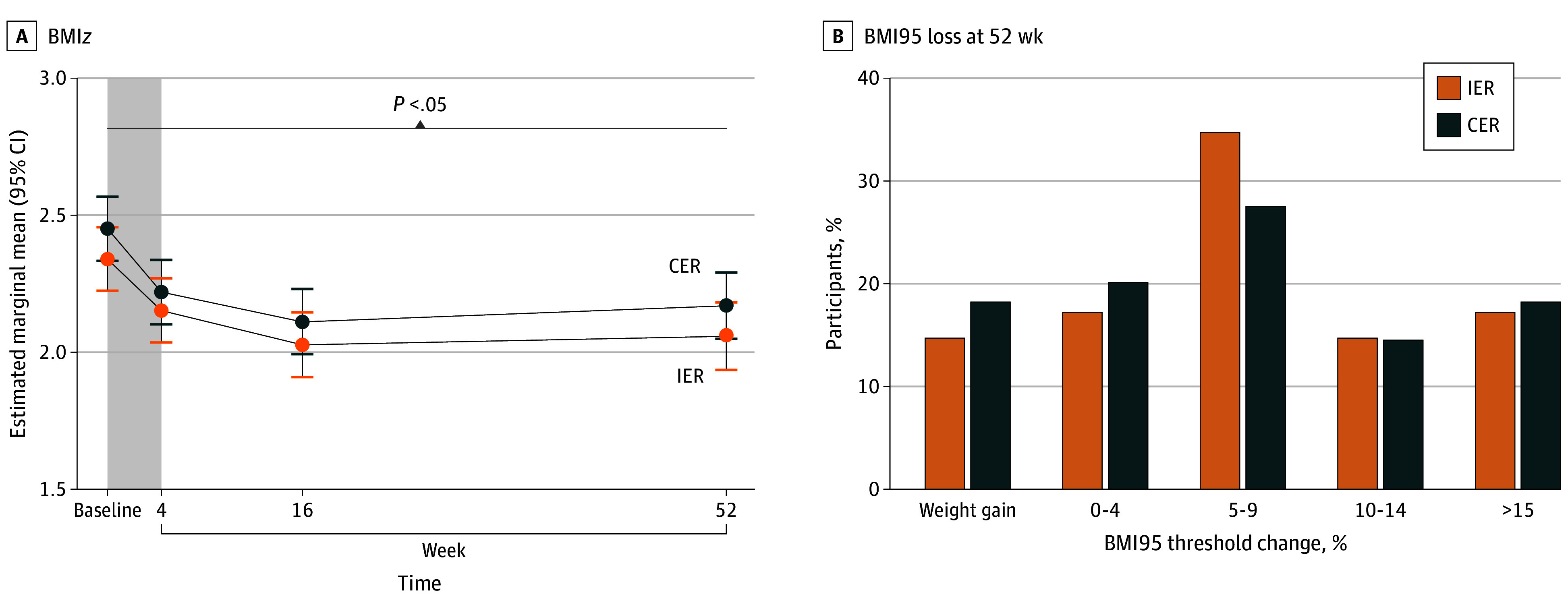
Change in Body Mass Index (BMI) *z* Score (BMI*z*) between Baseline and Week 52 and BMI Expressed as a Percentage of the 95th Percentile (BMI95) at Week 52 Compared With Baseline For BMI*z*, data are from 141 adolescents. The shaded area represents the 4-week very low-energy diet phase of the trial. Error bars indicate 95% CIs. For BMI95, data are from 94 participants who had BMI data at week 52. Body mass index was calculated as weight in kilograms divided by height in meters squared. CER indicates continuous energy restriction; IER, intermittent energy restriction.

**Figure 3.**
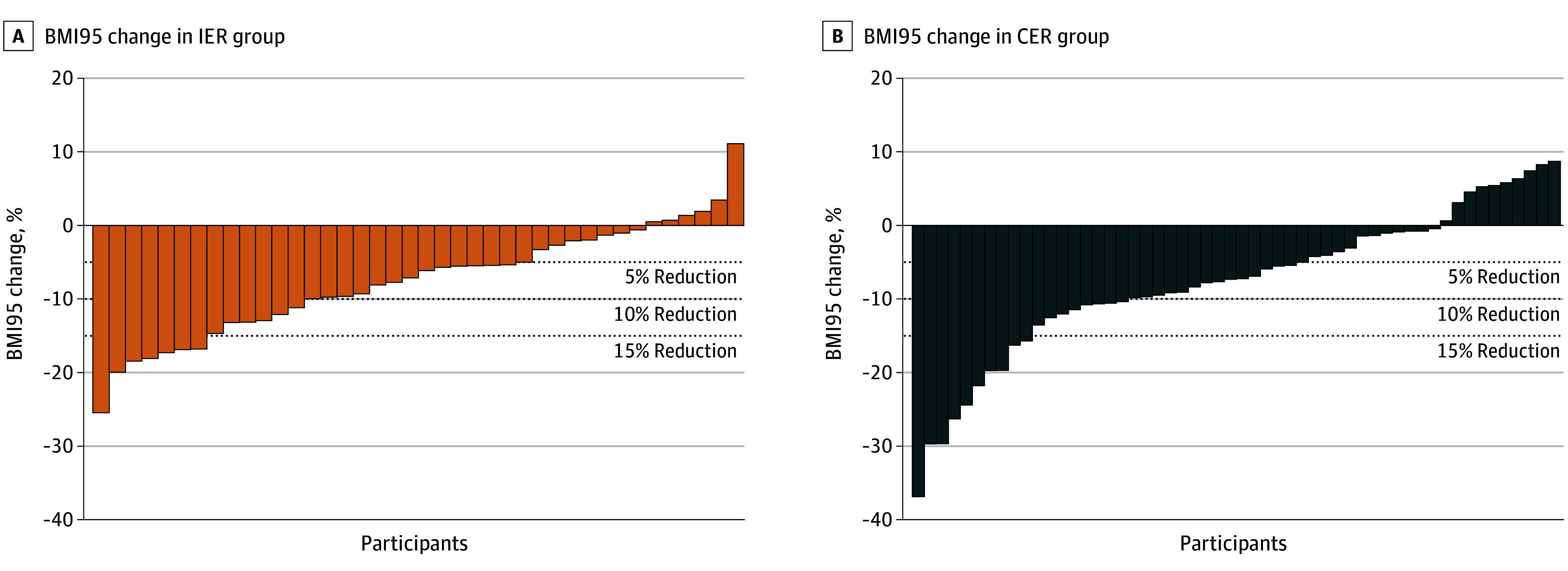
Change in Body Mass Index Expressed as a Percentage of the 95th Percentile (BMI95) at Week 52 Compared With Baseline For the intermittent energy restriction (IER) group, data are from 40 participants with BMI data at week 52; for the continuous energy restriction (CER) group, data are from 54 participants with BMI data at week 52. Body mass index was calculated as weight in kilograms divided by height in meters squared.

At week 52, the estimated marginal mean changes for BMI*z* were −0.28 (95% CI, −0.37 to −0.20) for the IER group and −0.28 (95% CI, −0.36 to −0.20) for the CER group (*P* < .001 for time, *P* = .502 for group × time); the estimated marginal mean changes for BMI were −1.62 (95% CI, −2.39 to −0.85) for the IER group and −1.53 (95% CI, −2.25 to −0.81) for the CER group; and the estimated marginal mean changes for BMI95 were −9.56 (95% CI, −12.36 to −6.83) for the IER group and −9.23 (95% CI, −11.82 to −6.64) for the CER group, with no differences between groups (Figure 2). The change in estimated marginal mean fat mass index was −1.59 (95% CI, −2.36 to −0.83) for the IER group and −1.45 (95% CI, −2.12 to −0.79) for the CER group (Table 2). The estimated marginal mean change in fat-free mass index was 0.13 (95% CI, −0.19 to 0.44) for the IER group and −0.05 (95% CI, −0.33 to 0.22) for the CER group. At week 16 there was a reduction in the estimated marginal mean fat mass index (−1.59 [95% CI, −2.35 to −0.83] for the IER group and −2.24 [95% CI, −2.71 to −1.77] for the CER group), which was maintained to week 52, with no difference between groups. At week 52, the proportion of participants who completed the study and achieved a 5% reduction in BMI95 was 35.0% (14 of 40) in the IER group and 27.8% (15 of 54) in the CER group. The proportion that completed the study and achieved a 10% reduction in BMI95 was 15.0% (6 of 40) in the IER group and 14.8% (8 of 54) in the CER group, and more than 15% reduction in BMI95 was achieved by 17.5% (7 of 40) in the IER group and 18.5% (10 of 54) in the CER group (Figure 3). Five participants in the IER group and 4 participants in the CER group met their goal weight. Two participants (1 in each group) who reached their goal weight did not attend the week 52 appointment.

**Table 2.** Estimated Marginal Means (SEs) for Outcomes for Each Intervention Group^a^

Outcome	IER				CER			
	Baseline	Week 4	Week 16	Week 52	Baseline	Week 4	Week 16	Week 52
BMI *z* score	2.34 (0.06)	2.15 (0.06)	2.03 (0.06)	2.06 (0.06)	2.45 (0.06)	2.22 (0.06)	2.11 (0.06)	2.17 (0.06)
BMI	34.83 (0.55)	33.09 (0.56)	32.23 (0.56)	33.21 (0.59)	35.95 (0.56)	33.79 (0.56)	33.03 (0.56)	34.42 (0.57)
BMI percentile	98.54 (0.34)	97.78 (0.34)	96.43 (0.36)	96.85 (0.40)	98.76 (0.34)	97.91 (0.35)	97.03 (0.36)	96.88 (0.37)
BMI95	127.84 (1.98)	121.07 (1.98)	117.05 (2.01)	118.24 (2.10)	131.61 (1.99)	123.39 (2.00)	119.75 (2.02)	122.38 (2.05)
Height, cm	167.3 (1.1)	167.3 (1.1)	168.2 (1.1)	169.5 (1.1)	169.0 (1.1)	169.2 (1.1)	169.7 (1.1)	171.3 (1.1)
Height *z* score	0.44 (0.13)	0.40 (0.13)	0.42 (0.13)	0.38 (0.13)	0.58 (0.13)	0.56 (0.13)	0.54 (0.13)	0.56 (0.13)
Waist circumference, cm	105.61 (1.50)	102.27 (1.51)	100.79 (1.55)	101.84 (1.68)	109.44 (1.52)	104.94 (1.53)	102.58 (1.55)	106.18 (1.59)
Waist to height ratio	0.63 (0.01)	0.61 (0.01)	0.60 (0.01)	0.60 (0.01)	0.65 (0.01)	0.62 (0.01)	0.61 (0.01)	0.62 (0.01)
FMI, kg/m^2^	15.95 (0.44)	NA	13.7 (0.45)	14.35 (0.50)	16.44 (0.45)	NA	14.20 (0.46)	14.99 (0.47)
FFMI, kg/m^2^	18.41 (0.23)	NA	18.12 (0.23)	18.53 (0.25)	18.95 (0.23)	NA	18.46 (0.23)	18.90 (0.24)
Body fat, %	46.12 (0.75)	NA	42.41 (0.78)	42.84 (0.86)	46.10 (0.76)	NA	42.87 (0.78)	43.32 (0.81)
Systolic blood pressure, mm Hg	118.8 (1.3)	115.0 (1.3)	114.3 (1.4)	115.3 (1.7)	120.3 (1.3)	113.7 (1.4)	115.0 (1.4)	117.9 (1.5)
Systolic blood pressure percentile	67.19 (3.26)	56.55 (3.31)	51.25 (3.53)	53.76 (4.26)	68.06 (3.28)	48.95 (3.41)	51.72 (3.50)	55.99 (3.73)
Diastolic blood pressure, mm Hg	67.2 (1.0)	66.1 (1.0)	64.9 (1.1)	65.6 (1.3)	69.0 (1.0)	63.7 (1.1)	65.1 (1.1)	64.8 (1.1)
Diastolic blood pressure percentile	67.19 (3.09)	52.67 (3.15)	50.69 (3.37)	53.64 (4.09)	68.06 (3.11)	43.41 (3.25)	51.75 (3.33)	55.98 (3.56)
Alkaline phosphatase, U/L	173.01 (10.08)	NA	159.06 (10.28)	135.90 (10.96)	169.15 (10.12)	NA	151.26 (10.22)	140.10 (10.51)
γ-Glutamyl transpeptidase, U/L	27.15 (2.04)	NA	23.21 (2.18)	26.93 (2.57)	26.40 (20.60)	NA	21.47 (2.14)	23.80 (2.29)
Aspartate aminotransferase, U/L	32.9 (1.95)	NA	27.9 (2.13)	30.3 (2.66)	34.1 (1.96)	NA	27.1 (2.08)	29.9 (2.31)
Alanine transaminase, U/L	41.41 (4.52)	NA	31.18 (5.00)	31.60 (6.12)	45.07 (4.58)	NA	27.34 (4.88)	28.27 (5.30)
Glucose, mg/dL	88.29 (0.90)	NA	87.39 (1.08)	87.57 (1.26)	89.73 (0.90)	NA	88.29 (0.90)	8955 (1.08)
Insulin, μIU/mL	26.83 (1.73)	NA	18.86 (1.87)	19.87 (2.16)	25.20 (1.73)	NA	17.85 (1.73)	19.44 (1.87)
Total cholesterol, mg/dL	174.90 (4.63)	NA	170.66 (4.63)	177.61 (5.02)	171.81 (4.63)	NA	164.09 (4.63)	168.34 (4.63)
Triglycerides, mg/dL	115.93 (7.96)	NA	98.23 (7.96)	103.54 (9.73)	132.74 (7.96)	NA	112.39 (7.96)	117.70 (8.85)
HDL-C, mg/dL	44.79 (1.16)	NA	45.17 (1.16)	45.17 (1.54)	44.02 (1.16)	NA	42.47 (1.16)	46.72 (1.54)
LDL-C, mg/dL	118.92 (4.25)	NA	116.60 (4.25)	123.55 (4.63)	116.99 (4.25)	NA	111.97 (4.25)	111.58 (4.63)
LDL:HDL ratio	2.82 (0.12)	NA	2.76 (0.13)	2.90 (0.14)	2.78 (0.13)	NA	2.73 (0.13)	2.56 (0.14)
Cardiometabolic profile, No. (%)		NA				NA		
Dyslipidemia, No. (%)	31 (43.7)	NA	16 (22.5)	17 (23.9)	29 (41.4)	NA	31 (44.3)	20 (28.6)
Elevated hepatic transaminases alanine transaminase and/or γ-glutamyl transpeptidase ≥1.5 times the upper limit of 30 U/L, No. (%)	24 (34.3)	NA	13 (23.2)	7 (18.9)	13 (18.8)	NA	10 (16.9)	8 (16.0)
Elevated fasting glucose of 100.90-124.32 mg/dL, No. (%)	5 (7.1)	NA	4 (7.1)	2 (5.6)	6 (8.8)	NA	5 (8.3)	9 (18.0)
Insulin resistance of fasting insulin-glucose ratio >20, No. (%)	52 (76.5)	NA	32 (57.1)	22 (61.1)	59 (86.8)	NA	31 (51.7)	30 (61.2)

Abbreviations: BMI, body mass index; BMI95, BMI expressed as a percentage of the 95th percentile; CER, continuous energy restriction; FMI, fat mass index; FFMI, fat-free mass index; HDL-C, high-density lipoprotein cholesterol; IER, intermittent energy restriction; LDL-C, low-density lipoprotein cholesterol; NA, not applicable (bone density scan and blood samples were not collected at week 4).

^a^ Data were included for 141 participants; means were estimated with an intention-to-treat analysis using a linear mixed model.

### Cardiometabolic Outcomes

Blood pressure percentiles and concentrations of total cholesterol, triglycerides, and fasting plasma insulin reduced over time (Table 2), but no difference was found between groups at any time. There were no differences in concentrations of HDL-C, low-density lipoprotein cholesterol, and fasting glucose levels across time points or between groups. At week 52 no differences were found in blood pressure status between groups. In total, 25 of the 90 participants (27.7%) who completed the study had persistent elevated blood pressure, 22 participants (24.4%) had resolution of elevated blood pressure, and 9 participants (10.0%) developed elevated blood pressure. The remaining 34 participants (37.7%) had normal blood pressure for the duration of the study.

Both groups had a reduction in the number of participants with insulin resistance (from 52 of 68 [76.5%] to 32 of 56 [57.1%] in the IER group and from 59 of 68 [86.8%] to 31 of 60 [51.7%] in the CER group) at week 16; however, at week 52, this was only observed in the CER group (from 59 of 68 [86.8%] to 30 of 49 [61.2%]). The occurrence of dyslipidemia for participants who completed the study was unchanged between baseline and week 52 (60 of 137 [42.6%] and 37 of 87 [42.5%], respectively), with improvement in the occurrence of impaired hepatic function tests (37 of 139 [27.0%] to 15 of 87 [17.2%], respectively). No differences were found in change of occurrence of dyslipidemia or impaired hepatic function between groups.

### Adverse Events

A total of 96 adverse events (in 67 of the 141 participants [47.5%]) were reported (eTable 3 in Supplement 2), with most occurring during phase 2. Most participants did not experience adverse events (50 of 71 [70.4%] in the IER group and 38 of 70 [54.3%] in the CER group). In the IER group, 13 participants (18.3%) experienced 1, and 10 (14.1%) experienced 2 to 6 adverse events. In the CER group, 21 participants (30.0%) experienced 1 adverse event and 12 (17.1%) experienced 2 to 5. The most reported adverse events were viral illness, including COVID-19 (34 of 96 [35.4%]), acute illness or injury unrelated to intervention (22 of 96 [22.9%]), and gastrointestinal disturbance (15 of 96 [15.6%] events across 3 IER group participants and 10 CER group participants). Eight events across 6 participants were classified as serious; 2 were possibly related to the intervention (1 participant developed gallstones and underwent subsequent cholecystectomy [CER group], and 1 developed atypical anorexia nervosa [IER group]). Two participants were withdrawn from the study by investigators due to mental health concerns.^22^

## Discussion

To our knowledge, this is the first randomized clinical trial to evaluate IER in adolescents. Adolescents with obesity-associated complications completed a 52-week intensive behavioral weight management program incorporating a VLED followed by IER or CER. After 52 weeks, significant reductions occurred in weight and some cardiometabolic outcomes compared with baseline in both groups. Occurrence of insulin resistance remained reduced in the CER group only at 52 weeks. No other differences in outcomes were found between the dietary intervention groups at any time point, and there was no difference in attendance. More adolescents withdrew from the IER group compared with the CER group due to not wanting to continue with that dietary pattern. However, completers tended to have more weight loss during VLED, as did those in the CER group, and the implications of this are not known. These results contrast with our hypothesis that IER would be more acceptable and lead to better weight loss compared with CER for adolescents with obesity-associated complications.

Previous randomized clinical trials in adults have demonstrated that IER is equally effective for weight and cardiometabolic outcomes when compared with CER. A 2018 systematic review in adults reported mixed findings when examining attrition, with some studies showing greater attrition in the IER group and others in the CER (control) group; however, most studies found no difference between groups.^15^ All studies in this review had intervention (weight loss) periods of less than 6 months.^15^ Indeed, our 6-month pilot study examining IER reported that adolescents with obesity liked the intervention, and given the opportunity at 12 weeks to continue IER or transition to a continuous energy diet, all (n = 23) chose to continue with IER.^16^ In the current study, most adolescents withdrew due to not wanting to continue with the study diet during phase 3 (16-52 weeks). Phase 3 was the study phase when contact with the study team was reduced compared with the earlier intensive phases. Hence, both the longer study duration and decreased contact with clinicians may explain the differences between this and the pilot study. Given that many participants engaged with the intervention during COVID-19, it is also possible that the IER diet was harder to maintain during lockdowns, when routines were potentially lacking. Nevertheless, no difference was found between the groups for weight and cardiometabolic outcomes, suggesting that IER may be offered to young people seeking an alternate dietary approach and result in similar improvements to physiologic health as CER.

Intensive behavioral weight management interventions are recommended by clinical practice guidelines.^5^ The 2023 American Academy of Pediatrics Clinical Practice Guideline recommends 26 hours of nutrition, physical activity, and behavior change lessons for 3 to 12 months, which is most effective when occurring face to face. Our intervention included 13 visits in 12 months, with additional support provided by text message, telephone, or email 9 times. In clinical practice, frequency and length of visits vary considerably,^32^ and most programs tend to be of lower intensity (ie, <25 contact hours).^34^ Kumar et al^33^ reported that, at 10 to 12 months, participants enrolled in a pediatric weight management program tended to have a larger reduction in BMI95 with at least 7 visits compared with those with 2 to 3 visits. In adults, the intensity of IER interventions range from weekly to monthly contact, with most studies ranging from 3 to 12 months.^15^ Our interventions were designed to meet the complex needs of adolescents with obesity,^36^ including using a motivational coaching model and specific strategies, such as social support, stimulus control, and monitoring for disordered eating and depression.^18^ Taken together, these findings suggest that for adolescent behavioral weight management, moderate to high intervention intensity is required for delivery of IER interventions, and interventions need to be formulated differently from those for adults. These findings are supported by guidelines and long-term data recommending ongoing support as a required component of adolescent weight management programs.^5^

Despite a lack of observed differences between groups, both intervention groups had reduced BMI compared with baseline at 12 months. Our findings are consistent with the 2017 Cochrane Review that compared behavior change interventions with control (6-24 months) and found a reduction in mean BMI*z* of −0.13 (95% CI, −0.21 to −0.05) and mean BMI of −1.18 (95% CI, −1.67 to −0.69).^38^ These findings contrast with adult data, which document weight regain between 6 and 12 months. Of note, the current study was conducted from 2018 to 2023, with most participants engaging in the intervention during the COVID-19 pandemic. Both study sites were affected by local lockdowns.^39^ Worldwide, there is consistent evidence of weight gain for adolescents and adults during the pandemic.^40^ Furthermore, there is emerging evidence suggesting that the effect of weight management interventions was blunted during COVID-19 lockdowns.^41^ Nonetheless, both groups achieved significant reductions in weight-related outcomes during the intervention. Future research is needed to determine long-term effects.

### Strength and Limitations

This study has several strengths. The behavioral weight management intervention was implemented by an experienced, multidisciplinary team. Adverse events were captured routinely, providing insights into the safety of intensive diets. Participants in our study were from culturally diverse backgrounds, enhancing the generalizability of our findings. However, the study also has some limitations. We failed to meet our estimated sample size due to COVID-19 lockdowns; however, we speculate that our observed effect would be unchanged with additional participants, given that we consistently observed no difference between groups across all variables and time points. Many dietitian support visits were conducted via tele-health, and the impact of this is unknown.

## Conclusions

The findings of this trial suggest that for adolescents with obesity-associated complications, IER can be incorporated into a behavioral weight management program. This treatment provides an option in addition to CER and offers participants more choice. Further data on risks and long-term outcomes beyond 12 months of intervention are required to confirm clinical implications.

## Notes

Supplement 1.
Trial Protocol and Statistical Analysis Plan

Supplement 2. **eMethods.** Statistical Analysis **eTable 1.** Protocol Changes Since Commencement of the Study **eTable 2.** Participant Demographics **eTable 3.** Adverse Events by Study Phase and Intervention Group

Supplement 3.
Data Sharing Statement
