# Supplementary material for: Intermittent Energy Restriction for Adolescents With Obesity: The Fast Track to Health Randomized Clinical Trial
Source: JAMA Pediatr. 2024 Aug 26:e242869. Online ahead of print. doi: 10.1001/jamapediatrics.2024.2869 (PMC11348084; doi:10.1001/jamapediatrics.2024.2869)
Supplement: Supplement 1. — Trial Protocol and Statistical Analysis Plan [file jamapediatr-e242869-s001.pdf]

Supplementary Online Content

Lister NB, Baur LA, House ET, et al. Intermittent energy restriction for adolescents with obesity: the Fast Track to Health randomized clinical trial. *JAMA Pediatr.* Published online August 26, 2024. doi:10.1001/jamapediatrics.2024.2869

eMethods. Statistical Analysis

eTable 1. Protocol Changes Since Commencement of the Study

eTable 2. Participant Demographics

eTable 3. Adverse Events by Study Phase and Intervention Group

This supplementary material has been provided by the authors to give readers additional information about their work.

© 2024 Lister NB et al. *JAMA Pediatrics.*

eMethods 1 - Statistical analysis

Linear mixed models with an autoregressive first order covariance structure and restricted maximum likelihood (REML) were used to estimate the change in outcomes between baseline, week-4, -16, and -52. Time (baseline, week-4, -16 and -52) was entered as a fixed (categorical) variable. Baseline was assumed to be equal across groups. The mixed models procedure accounted for within-participant correlations and missing data points. A time by group interaction was included to investigate whether rates of change in outcomes were different between groups. Assumptions of modelling were met, and results are presented as the difference in estimated marginal means (EMM). A post-hoc pairwise comparison was conducted to compare means over time. Analyses were repeated using an unstructured covariance model as part of sensitivity analyses. McNemar's chi square test was used to assess change between baseline and week-52 for categorical data. Independent sample t-tests were used to test for differences in the primary outcome at baseline, week-4 and -16 between completers and non-completers.

© 2024 Lister NB et al. *JAMA Pediatrics.*

eTable 1 – Protocol changes since commencement of the study

Version Number	Modifications	Date of HREC approval
2	• Addition of support phone call during week-1<br>• Addition of method for measuring resting energy expenditure (REE) at Monash	31.10.17<br>*(Prior to first participant enrolment)*
2.1	• Removal of blood collection at week-4<br>• Addition of very low energy diet acceptability questionnaire at week-4	20.2.18<br>*(before first participant reached Week 4)*
2.2	• Additional REE measurement added at week-4<br>• Provision of Fitbit at week-16	21.5.18
2.3	• Addition of intervention fidelity protocol (including use of CARE questionnaire and semi-structured interviews with participants and parents/guardians) (PhD sub-study)<br>• Addition of weight maintenance procedures for each study arm for participants who reach their goal weight	17.10.18
2.4	• Addition of participant follow-up at 24 months (including additional consent to contact families at 24 months)	30.01.19
2.5	• Inclusion of clinical psychologist support including for review of screening surveys, liaising with other study clinicians and clinical assessment of 'at risk' participants<br>• Inclusion criteria updated to include requirement for participants to have at least one cardiometabolic complication	03.04.19
2.6	• Addition of gene expression analysis at Monash (PhD sub-study)<br>• Details of participant management at study completed added, including engagement of referral services and transition to weight maintenance	30.07.19
2.9	• Details of the study Oversight Committee added*<br>**This committee consisted of independent clinical monitors, a statistician and trial investigator (non-voting role). They were responsible for identifying safety concerns and making recommendations to the Trial Steering Committee for continuing or stopping the trial. The trial would be stopped if >5% participants overall were withdrawn by investigators for safety reasons related to the intervention. This would include any significant adverse events (SAEs) that occur.*	12.02.20
3	• Full 24-month follow-up procedures added, including the option for on-site review and/or completion of online questionnaires	14.04.20
3.1	• Use of telehealth platforms (previously limited to Zoom, Skype and FaceTime) updated to include platforms approved by the health facility.<br>• COVID-19 clause added to specify that face-to-face appointments will occur in line with hospital directives.	13.09.21

© 2024 Lister NB et al. *JAMA Pediatrics.*

3.2	• Procedures were updated to stipulate that appointments following the transition to weight maintenance or any additional reviews (for clinical or psychological reasons) should occur face-to-face and not via telehealth	28.07.22

© 2024 Lister NB et al. *JAMA Pediatrics.*

eTable 2– Participant demographics

	IER (n=71)	CER (n=70)	All Participants (n=141)
Country of birth			
- Australia	58	59	117
- India	4	2	6
- Vietnam	2	0	2
- Other	4	5	9
Mother country of birth			
- Australia	44	46	90
- Europe	3	4	7
- Middle East/ North Africa	8	8	16
- Asia	11	6	17
- Pacific	1	3	4
- Americas	1		1
- Others	2	1	3
Father country of birth			
- Australia	41	37	78
- Europe	1	3	4
- Middle East/ North Africa	9	14	23
- Asia	12	5	17
- Pacific		5	5
- Americas	1		1
- Others	3	1	4
Language spoken at home			
- English	68	63	131
- Mandarin		1	1
- Arabic	10	14	24
- Cantonese		1	1
- Vietnamese	3		3
- Italian		2	2
- Greek	3	1	4
- Spanish	1	1	2
- Hindi	1	2	3
- Dari	2	1	3
- Punjabi	1		1
- Other	8	5	13
Mother Aboriginal / Torres Strait Islander			
- Aboriginal	1		1
- Torres Strait Islander			
- Neither	42	46	88
Father Aboriginal / Torres Strait Islander			
- Aboriginal	1		1
- Torres Strait Islander			
- Neither	40	37	77
Parental marital status			
- Single parent	17	23	40
- Married/ de facto	53	45	98
Mother education level			

© 2024 Lister NB et al. *JAMA Pediatrics.*

- Some high school	10	6	16
- Completed HSC/Leaving/Year 12	4	13	17
- Any further education	51	46	97
- Other	5	3	8
Father education level			
- Some high school	11	12	23
- Completed high school	8	8	16
- Any further education	43	42	85
- Other	6	2	8
Mother employment			
- Working full-time	33	28	61
- Working part-time	14	18	32
- Unemployed / home duties	16	20	36
- Student	3	1	4
- Retired	1		1
- Permanently unable to work/ ill		1	1
- Other	2		2
Father employment			
- Working full-time	53	47	100
- Working part-time	5	2	7
- Unemployed/ home duties	1	3	4
- Student	1	1	2
- Retired	2		2
- Permanently unable to work/ ill	4	3	7
- Other	1	5	6
Household income (Australian dollars)			
- Less than $20,000	4	7	11
- $20,000 - $40,000	3	5	8
- $40,000 - $60,000	3	9	12
- $60,000 - $80,000	15	9	24
- More than $80,000	28	23	51
- Prefer not to say	16	12	28
SEIFA (from postcode)			
Index of Relative Socio-Economic Disadvantage (IRSD)			
- Greater than 75%	22	22	44
- 50% - 75%	19	14	33
- 25% - 50%	9	12	44
- Less than 25%	20	20	40
Index of Relative Socio-Economic Advantage and Disadvantage (IRSAD)			
- Greater than 75%	25	25	50
- 50% - 75%	18	14	32
- 25% - 50%	12	13	25
- Less than 25%	15	16	31
Parent with obesity	46	51	97
Family history of diabetes	56	61	117

© 2024 Lister NB et al. *JAMA Pediatrics.*

eTable 3 – Adverse events by study phase and intervention group

	All Participants (n=141)	IER (n=71)	CER (n=70)
Total	96[a]	42	54
- Phase 1	2	2	0
- Phase 2	60	24	36
- Phase 3	32	15	17

[a]Two participants (one IER group; one CER group) reported a having COVID19 infection during the study at their final visit but could not recall dates/study phase of the event.

[b]Eight events across six participants were classified as serious adverse events.

© 2024 Lister NB et al. *JAMA Pediatrics.*
