# Supplementary material for: Intermittent Energy Restriction for Adolescents With Obesity: The Fast Track to Health Randomized Clinical Trial
Source: JAMA Pediatr. 2024 Aug 26:e242869. Online ahead of print. doi: 10.1001/jamapediatrics.2024.2869 (PMC11348084; doi:10.1001/jamapediatrics.2024.2869)
Supplement: Supplement 3. — Data Sharing Statement [file jamapediatr-e242869-s003.pdf]

Data Sharing Statement

Lister. Intermittent Energy Restriction for Adolescents With Obesity. *JAMA Pediatr*. Published August 26, 2024. doi:10.1001/jamapediatrics.2024.2869

Data
Data available: No

Additional Information
Explanation for why data not available: Data may be shared subject to further ethics approval.
